# Phenotyping of Adaptive Immune Responses in Inflammatory Diseases

**DOI:** 10.3389/fimmu.2020.604464

**Published:** 2020-11-25

**Authors:** Jens Y. Humrich, Joana P. Bernardes, Ralf J. Ludwig, David Klatzmann, Alexander Scheffold

**Affiliations:** ^1^ Department of Rheumatology and Clinical Immunology, University Hospital Schleswig-Holstein—Campus Lübeck, Lübeck, Germany; ^2^ Institute of Clinical Molecular Biology, Christian-Albrechts-University Kiel, Kiel, Germany; ^3^ Lübeck Institute of Experimental Dermatology (LIED), University of Lübeck, Lübeck, Germany; ^4^ Sorbonne Université, INSERM, Immunology-Immunopathology-Immunotherapy (i3), Paris, France; ^5^ Institute of Immunology, Christian-Albrechts-University Kiel, Kiel, Germany

**Keywords:** immunophenotyping, sequencing, proteomics, inflammation, precision medicine, TCR repertoire, antigens

## Abstract

Immunophenotyping on the molecular and cellular level is a central aspect for characterization of patients with inflammatory diseases, both to better understand disease etiopathogenesis and based on this to develop diagnostic and prognostic biomarkers which allow patient stratification and tailor-made treatment strategies. Technology-driven developments have considerably expanded the range of analysis tools. Especially the analysis of adaptive immune responses, often regarded as central though mostly poorly characterized disease drivers, is a major focus of personalized medicine. The identification of the disease-relevant antigens and characterization of corresponding antigen-specific lymphocytes in individual patients benefits significantly from recent developments in cytometry by sequencing and proteomics. The aim of this workshop was to identify the important developments for state-of-the-art immunophenotyping for clinical application and precision medicine. We focused here on recent key developments in analysis of antigen-specific lymphocytes, sequencing, and proteomics approaches, their relevance in precision medicine and the discussion of the major challenges and opportunities for the future.

## Introduction

Immunophenotyping is a key method for monitoring and diagnosing of immunological diseases, such as infectious diseases, chronic inflammatory and autoimmune diseases and allergy. For many of these the actual disease cause is still elusive or as in the case of COVID-19 the origin and contribution of potentially pathogenic immune reactions to severe disease. Classically immune monitoring is accomplished *via* measurement of serum biomarkers, including acute phase proteins, serum cytokines, or antigen-specific serum antibodies. However, a deeper understanding of the underlying disease cause and the pathogenic mechanisms, requires the identification and characterization of the responsible immune cells and their interaction partners. Most of these diseases are restricted to specific organs or antigens and there is plenty of, though mostly indirect, evidence that antigen-specific lymphocytes are the pathogenic drivers of disease. However, due to technological restrictions these cells are barely characterized and in many inflammatory and autoimmune diseases the target antigens of the pathogenic immune reaction are still unknown. This is a major roadblock for development of personalized medicine, that is disease- and patient-specific diagnostics and therapies targeting the disease cause rather than the symptoms.

Thus a major aim of immunophenotyping in the age of personalized medicine, diagnostics and therapy is the development of tools allowing to dissect the immune response on the level of antigen-specific T and B cells, their relevant interaction partners and effector molecules. During a workshop on immunophenotyping in inflammatory diseases, which took place at the international symposium of the Cluster of Excellence “Precision Medicine in Chronic Inflammation (PMI)” of the universities of Kiel and Lübeck on February 18^th^ 2020 in Hamburg, Germany, recent developments facilitating such antigen-specific approaches and beyond were discussed.

## State-of-the-Art and Key Developments

### Flow-Cytometry and Analysis of Antigen-Specific Lymphocytes

The method of choice for detailed characterization of immune cell composition and phenotype is flow-cytometry, which does not only allow rapid quantitation of the main leukocyte subsets but also their detailed phenotypic characterization, including the differentiation of the complex cosmos of lymphocyte subpopulations at a single cell level. A state-of-art overview of currently available cytometric technologies can be found under (1). Instrumental to this has been the steadily increasing numbers of parameters, which can be measured from individual cells. Theoretically > 40 parameters per cell are currently possible, although this is still not part of the clinical routine. However, recent developments especially of multi-laser instruments or spectral cytometers have significantly improved the ease of use, especially for the combination of various fluorescent dyes within multiplex panels (1–4). This may bring multi-parameter flow-cytometry closer to the clinical diagnostic lab reality.

**Figure 1:**
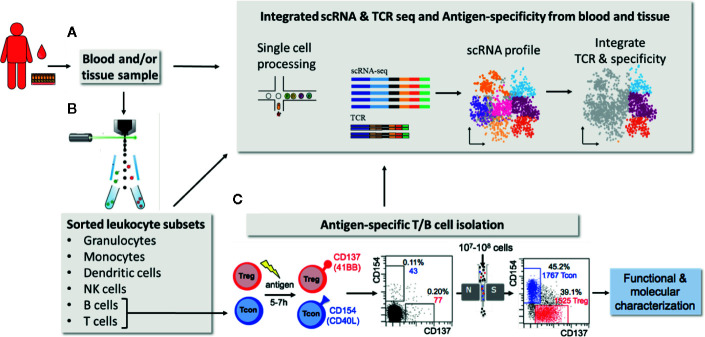
Integrated scRNA & TCR seq and antigen-specificity from blood and tissue. (Single cell) RNAseq provides unique multi-parameter information of single cells **(A)** or sorted cellular subsets **(B)**. Further the combination of antigen-receptor sequencing with scRNASeq allows correlation of RNA expression with clonal lymphocyte populations **(A)**. In addition, antigen-receptor sequences derived from antigen-specific lymphocytes of the same donor, obtained *via* combined magnetic pre-enrichment and fluorescence based flow-sorting, allows to integrate antigen-specificity and certain functional aspects **(C)**. TCR/BCR information from antigen-specific lymphocytes may also be used to track antigen-specific cells within small tissue samples.

Identification of antigen-specific lymphocytes is a challenge due to their low frequency and the availability of antigen-reagents allowing direct labeling of their antigen-receptor (5, 6). Although fluorescently tagged antigens can in principle be used to directly label B cells these are still no routine reagents and staining procedures (1). Even more challenging the exact peptide/MHC combinations need to be defined to generate the recombinant peptide/MHC-multimers for antigen-specific T cell staining. This is a significant difficulty especially for the analysis of human T cells due to the high HLA-diversity in general and in particular for CD4+ T cells due to low quality MHC class II reagents, complex antigens or pathogens and/or unknown peptide targets. Therefore especially human CD4+ T cell analysis is mainly based on functional read-outs, following *in vitro* stimulation with antigenic peptides, proteins or protein extracts. Several technologies to detect antigen-reactive T cells have been developed (1, 5, 6). A common problem for all antigen-specific assays is the low frequency of target cells. A frequent solution to this is the *in vitro* expansion of rare T and B cells prior to analysis. However, this introduces an unpredictable bias with regard to phenotype, function and selection of certain cell subsets. Direct approaches utilizing minimal stimulation times (5–7 h) combined with powerful rare cells enrichment strategies, e.g., *via* magnetic cell separation, provide a more direct view (7–9). Rapid induction of CD154 expression in all conventional T helper cells (Tcon) (10, 11) and of CD137 in Tregs (12) within a short time window of 5–7 h after *in vitro* antigen stimulation has proven to be highly sensitive and specific to identify essentially all reactive CD4+ T cells. This can be combined with magnetic enrichment strategies, termed antigen-specific T cell enrichment (ARTE) (7). This technology has been used to characterize human CD4+ T cell responses in a number of clinical settings, including allergy (13), IBD (13, 14) and infectious diseases (15). The unique strength of direct enrichment-based approaches is the access to highly purified antigen-specific T cells, e.g., *via* fluorescence-based flow-sorting technologies, which is ideal for combination with downstream “-omics” technologies, allowing their deep molecular profiling as well as functional analyses in unparalleled resolution (13, 14, 16) (**Figures 1B, C**). This includes TCR sequencing to determine T cell clonality and/or TCR affinity measurements (13, 14, 16) (**Figures 1A, C**). The particular relevance of activation-based assays has been highlighted by recent data on SARS-CoV-2-specific T cells which allowed rapid identification of target proteins and overall T cell reactivity in patients (17–19) as well as clonality and affinity differences between mild and severe COVID-19 (20). In general B/TCR sequencing provides unique insight in B and T cell composition as outlined below. However, it has to be emphasized here, that the combination of antigen-specific B/T cell sorting with antigen-receptor sequencing is particularly powerful. Antigen-receptor sequences from cells with known specificity can be used as molecular identifiers to track antigen-specific clones in samples from the same patient. This has been used, for example, to track the modulation of antigen-specific T cells in bulk TCR sequencing data from patients with multiple sclerosis during pregnancy (21). Similarly, known TCR sequences from paired blood and tissue samples will allow identification of antigen-specific T cells from bulk or single cell RNA sequencing data. The latter approach may allow to follow and deeply characterize antigen-specific T cells even in small tissue samples.

### TCR Repertoire Analysis

Clonal expansion is one of the hallmarks of adaptive immune responses. Therefore T cell receptor (TCR) analyses carry great potential for investigating the specificity of the T cell immune response under physiological and pathophysiological conditions. There are however multiple issues to consider in advance. (i) The first is about numbers. As outlined above antigen-specific lymphocytes are rare. From the possible 10^19^ theoretically possible combinations (22), TCR selection during thymopoiesis selects for each individual a repertoire of approximately 10^7^ to 10^8^ unique TCRs (23). Thus looking for specific TCRs, even expanded ones, resembles searching for a needle in a haystack. (ii) The second is about sampling: where, and how much? Indeed, how much of the immune response targeting a pulmonary infection is represented in 10^5^ cells from blood? (iii) The third is about T cell functional diversity. As an example, since Treg and effector T cells have opposite effects, what would mean the identification of a given TCR if you cannot assign it to one or the other cell population? (iv) The fourth is about technology: TCRs are made of alpha and beta chains that cannot (accurately) be paired by knowledge. Only single-cell sequencing can provide paired chains. Also, the accuracy of the technology that heavily relies on PCR amplification and its capacity to detect rare clonotypes might affect results (24). Despite all these limitations, TCR sequencing carries enormous potential in particular in combination with cytometric techniques discussed above. There are currently two major approaches to TCR studies: (i) global TCR repertoire assessment from a large number of cells, classically applied to study peripheral blood T cells, and which can generate millions of alpha and beta unpaired TCRs per sample, and (ii) single-cell sequencing of smaller samples, such as tissue infiltrating cells, which generates in the order of 10^3^-10^4^ clonotypes. Global TCR studies are providing much larger repertoires, from which the challenge is to identify the relevant TCRs. Numerous algorithms have been and are still developed to extract relevant information, such as repertoire diversity, expansions of rare cells and cross-reactivity (25–30). They, for example, allowed to identify the TCRs responding to vaccination (31) and in our experiments identified TCRs are specific and even predictive of given autoimmune diseases (David Klatzmann, unpublished observations). In contrast, single-cell studies have the advantages to relate TCRs directly to a setting/tissue or an ongoing immune response and to provide their paired alpha and beta chains (32). Noteworthy, it also allows to concomitantly investigate, (i) the binding of dextramers or MHC-multimers to give clues on the cell specificity, and (ii) the single-cell transcriptome to provide, for example, the activation status of cells with specific TCRs (32, 33).

Bulk and single cell TCR sequencing are truly complementary and even synergistic. Bulk TCR sequencing allows to provide general information on the repertoire, with indices reflecting its diversity and “normality” (25–30). Also, appropriate algorithms can identify thousands of TCRs of potential interest, based for example on their frequency in the sample compared to their theoretical probability of generation (34). The relevance of these sequences can further be explored by blasting them with databases of TCRs with known specificities (35, 36). On the other hand, single-cell sequencing, despite its limitation in terms of the number of sequences, has the advantage of providing the paired sequences in link to a specific setting. For example, the T cells present in bronchoalveolar lavages of patients with lung infection (33) or the cerebro-spinal fluid of patients with neurogenerative diseases can reasonably be assumed to be part of the ongoing immune reaction associated with the condition. Thus, as a recommendation, we would suggest the following:

- To sequence the bulk repertoire of at least 10^5^ purified/enriched T cell populations, at least always separating Treg from Teffs, and using a robust method (24).
- To refrain from performing single-cell TCR sequencing from peripheral blood except if relevant subsets can be enriched, i.e., by antigen-specific cytometry assays as described above.
- To perform single-cell TCR sequencing on samples originating from a small cell population, whether this comes from their location or their expression of relevant markers.

Besides, one should make sure to analyze the obtained datasets with experts of the field and not forget to make their datasets public such as not to waste precious and costly results (37). Indeed, even if we could analyze the entire TCR repertoire of billions of humans bearing each 10^12^ T cells, we would still be far from approaching the 10^61^ different sequences that theoretically could be produced by the T cell machinery (38). Thus, any TCR sequence that was actually produced (i.e., detected in a sample) had dramatically much smaller chances to appear that the reader has to win the lottery, and thus represents extremely valuable knowledge (from theory to reality) that must be memorized.

### RNA Sequencing

High-throughput sequencing methods have been the key to disentangle immune phenotypes of inflammatory diseases for decades. Advances in mapping disease activity, identification of early disease, and therapy-related molecular markers have been made possible by the widespread methodology of bulk RNA-seq, a robust and nowadays inexpensive method of sequencing the transcriptome. Despite the effort, the current number of direct applications of molecular-based disease diagnostic and treatment remains limited (39). One explanation for the poor output of bulk RNA-seq-driven methods is that it solely depicts the transcriptome of a homogeneous and unidentifiable agglomerate of different cell types. Thus, masking cell-specific transcription changes and neglecting the impact of cell type proportions in diseased tissues. Moreover, skewed levels of expression of molecular markers are expected when researchers focus on the immune compartment and particular aberrant proportions in inflamed tissues (40–42).

A possible approach to overcome the lack of cell-specificity is the implementation of single-cell sequencing (scRNA-seq), which generates a transcriptional landscape by cell type. This methodology is capable of acquiring up to 80,000 cells per run, enabling unprecedented power to study patterns of expression of even rare cell types linked to inflammatory diseases (43). Moreover, researchers can customize cell type identification based on their need, by using cell-specific signature genes (e.g., *MKI67* high expression for proliferative lymphocytes) coupled with numerous reference transcriptomic datasets (44). Recent technological advances have also provided access to new layers of information by merging cell-specific expression with TCR or BCR information (45, 46) and one can even produce large-scale immunophenotyping panels of tens to hundreds of antibodies (47). This type of analysis leads to the unbiased classification of T or B cell populations of interest and the relationship TCR or BCR with the respectively cell-specific transcription. However, this methodology not only comes with a very high price tag but also with its own set of challenges [for review please see (48)]: (*i*) scRNA-seq only represents a subset of tissue, thus rare cell-types, such as antigen-specific lymphocytes might be missed by chance if the number of cells acquired is not suitable (49). (*ii*) Experimentally, the methods are prone to batch effects, e.g., time from acquiring sample to sample preparation is inversely correlated with sample quality and stability of expression data (50), different technologies and protocols may also acquire different cell proportions and gene counts (51). (*iii*) Despite the robust methods available for PBMCs, dissociation of fragile tissues such as the brain or intestinal epithelium, might be problematic and carry on skewed assessments of the tissue (52). (*iv*) Specific to TCR applications, the reads might be aligned to an incomplete “reference” genome that might also be biased by somatic rearrangements and mutations (45).

Regardless of the methodological pitfalls, crucial advances in immune profiling and particularly in mapping inflammatory diseases have been pinned to scRNA-seq technology: by integrating scRNA-seq and antigen-receptor sequencing (**Figure 1A**), Boland et al. have contributed to resolving clonal relationships of the PBMCs and the intestinal mucosa in ulcerative colitis by coupling an enrichment for IgG1+ in plasma cells in the inflamed tissue with a specific γδ T cell subset in the peripheral blood of patients (53). This publication elegantly highlights how combining gene expression data paired with receptor sequence enables re-expression and testing for antigen binding and function of potentially relevant molecular markers. Taken all together, scRNA-seq has quickly become the go-to technology for immune profiling of individual cells on a large scale.

### Proteomics Approaches

In addition to multicolor flow-cytometry and transcriptomics, proteomics of sorted cell subsets and various tissues allow to provide detailed insights into pathology-associated changes in complex immune-mediated diseases (54). There are a multitude of proteomic techniques that can be used to analyze changes of protein expression; e.g., mass spectrometry-based proteomics (55, 56). This, however, requires a high degree of specialization and access to respective core facilities. Thus, we here focus on proteomic techniques that can, in principle, be applied in most laboratories: (i) Bead-based multiplex arrays for flow cytometry to determine cytokine concentrations, (ii) protein arrays for autoantibody profiling, (iii) proximity extension assay proteomics, (iv) multi-parameter immunohistochemistry, and (v) determination of kinase activity.

Bead-based multiplex arrays for flow cytometry have become a standard technology for the fast and high-throughput analysis of cytokine concentrations in biological samples, such as blood or cell culture supernatants (57, 58).

Several companies offer auto-antigen protein arrays that allow the simultaneous detection of multiple autoantibodies from human serum samples, including the immunoglobulin subtype differentiation (59). These led to the identification of autoantibodies in diseases that are currently not considered as autoimmune diseases, e.g., chronic obstructive pulmonary disease (60) or neuropsychiatric diseases (61). Furthermore, autoantibody arrays can be used to identify additional autoantibody reactivity beyond known autoantibodies, for example in pemphigus (62, 63). However, the mere detection of autoantibodies in any given population is usually not informative because autoantibodies can be detected at high frequency also in healthy blood donors (64, 65) and, are even present in IVIG preparations (66) used to treat autoimmune diseases (67). Thus, in depth characterization of their functions and of the corresponding B and T cells is mandatory.

Proximity extension assay (PEA) proteomics allow to identify up to 1500 human proteins in any solution. The assay is optimized for serum, but use of lysed tissue extracts has also been described (68, 69). For PEA, samples are incubated with a pair of oligonucleotide-labeled antibodies binding to different epitopes of a specific protein. When these antibodies are in close proximity, they hybridize, and are extended by a DNA polymerase. This unique DNA barcode is then amplified by PCR, whereby the amount of each specific DNA barcode in quantified by PCR (70). Regarding mouse samples, a limited number of proteins (90–100) can be analyzed using this technique. However, up to now, the majority of published reports on PEA have investigated patients with chronic inflammatory skin conditions, but not with systemic inflammatory or autoimmune diseases (69, 71–74). Based on our own experience, we believe that the PEA technology will increasingly be used to study alterations of proteomic signatures in chronic inflammatory diseases, and thus will significantly contribute to the understanding of disease pathogenesis. PEA also offers the possibility to stratify certain patients according to their proteomic signature, and/or allow to predict treatment responses.

While all of the above technologies are primarily used to identify proteomic signatures in blood, multi-parameter immunohistochemistry enables to determine protein expression in tissues. One platform is the multi-epitope ligand cartography (MELC) robot technology that has been described in 2006 (75). The principle behind MELC is repeated staining and subsequent bleaching of histology specimens with automated image recording. Interestingly, only few papers have been published using this technique. Overall, we believe that multi-parameter immunohistochemistry is a potentially powerful tool to understand both protein expression and location, however, may not be suitable for routine diagnostics due to high costs and low sample throughput.

Aberrant kinase activation, in particular of the Janus Kinase (JAK) family, has been identified as a therapeutic target for an increasing number of autoimmune diseases (76–78). A relatively novel method to determine kinase activity in cell culture, as well as tissue or blood samples, is a peptide microarray, commercialized under the name of PamGene (79). In this assay, either serine or threonine-containing peptides are spotted on a microarray. After application of the cell or tissue homogenates, kinases within these phosphorylate their respective substrates. Substrate phosphorylation is detected over time using specific antibodies. Based on the phosphorylation pattern of substrates, the activity of kinases can be determined. This peptide microarray for detection of kinase activity can, among others, be used for the identification of novel therapeutic targets (80, 81), discovery of biomarkers (82, 83), and understanding of physiological immune functions (84). The main focus of the PamGene has so far, however, been in the field of cancer research. Based on our own experience with this technique, we expect that this microarray technique will significantly contribute to our understanding of the pathogenesis of chronic inflammatory and autoimmune diseases in the near future.

## Perspective

Despite significant progress in translational and personalized medicine in the last decade, there is still an urgent need to identify disease-specific signatures and individual biomarkers allowing patient stratification, prediction of responses to therapy including the assessment of dynamic changes and the identification of novel therapeutic targets. In addition, it should be mentioned that data validation and knowledge transfer into clinical routine is often missing in the majority of applied approaches. Modern high-throughput technologies can be extremely useful and time saving in immunological research and become increasingly relevant especially for precision medicine, but are also highly cost intensive. Thus, the right method or combination of methods suitable to adequately address the questions of your study needs to be carefully selected to get a reasonable balance between costs and benefits. On the other hand one has to consider in advance which relevant information and conclusions can be extracted from multi-parameter data sets and how these data can be combined and integrated in a biological and clinical context. The involvement of experienced system biologist and biostatisticians in the planning of a study is therefore highly recommended in order to get most information from the applied methods.

## Author Contributions

JH, JB, RL, DK, and AS equally contributed to this review according to their area of expertise and with regard to content and structure of the manuscript. All authors contributed to the article and approved the submitted version

## Funding

This work was supported by German Research Foundation grants EXC2167.

## Conflict of Interest

The authors declare that the research was conducted in the absence of any commercial or financial relationships that could be construed as a potential conflict of interest.
